# Monkey pox epidemic in Democratic Republic of Congo needs urgent attention

**DOI:** 10.4314/ahs.v25i3.6

**Published:** 2025-09

**Authors:** Nida Shoaib, Maryam Saghir, Hozefa Mateen

**Affiliations:** Jinnah Sindh Medical University

**Keywords:** Monkey pox, democratic republic of Congo, Skin Diseases

The virus that causes mpox, formerly known as monkeypox, is a member of the Ortho-poxvirus genus within the Poxviridae family. Other viruses like smallpox and cowpox are also related^1^. The virus presents with fever, swollen lymph nodes, and an excruciating rash. Furthermore, Mpox has two primary genetic variants or clades: Clade I (found primarily in Central Africa) and Clade II (found mainly in West Africa). Additionally, Clade II has further sub-divisions, including Clade IIb, which has dominated in the recent global outbreak. Mpox is a medical condition that can lead to severe complications such as bacterial infections, pneumonia, vision loss, severe gastrointestinal issues and inflammation of various organs, including encephalitis (inflammation of the brain) and myocarditis (inflammation of the heart).

Moreover, in extreme cases, it can be fatal, particularly among immuno-compromised HIV-positive men. There is a high risk of rapid transmission of monkeypox cases in areas of extreme population density and sexual violence, such as in the camps of displaced people around the capital of North Kivu, Goma. Additionally, the mass population movements taking place within and outside of DRC pose a real threat to the spread of this disease. However, the main concern is that patient care and monitoring remain as limited resources in the DRC. On the other hand, the lack of vaccinations further aggravates the situation.

Only 24% of suspected pox cases are being tested in DRC due to a limited testing capacity^2^. A highly positive rate of 62% is observed, with most of the cases remaining unconfirmed and are diagnosed solely based on the symptoms. Furthermore, there is a misconception in some countries that the disease is associated with witchcraft, which further complicates its adherence to public health measures.

Mpox has been sporadic in Africa since 1970. However, it re-emerged in Nigeria in 2017 and spread to Europe, America, and globally in 2022, with 110 countries reporting about 87,000 cases and 112 deaths. The Mpox outbreak was also recorded in refugee camps in Sudan. The epidemic has predominantly affected men who have sex with men through sexual networks. In contrast to the data collected over the past years, the number of cases in 2024 has already surpassed historical totals, indicating a concerning increase^3^ As of June 2024, the WHO had received reports of 99,176 confirmed cases, 535 probable cases, and 208 deaths, amongst which 96.4% of the cases (with the median age 34) are male. The DRC has experienced a dramatic increase in the number of cases and deaths in 2024, with significant outbreaks being reported from 23 provinces (Equateur, Sud Ubangi, Sankuru, and South Kivu).

From January 2022 to June 2024, 30,711 suspected cases and 1326 deaths have been reported in the DRC^1^. In August 2024, 15,664 suspected cases and 548 deaths were reported to the CDC^4^. Children under the age of 5 are the most affected group, constituting about 39% of reported cases and 62% of deaths among this group. Subsequently, mpox can also spread through close contact with humans or animals that are afflicted with the disease. Furthermore, the epidemic is spreading at an alarming rate due to a new genetic mutation (clade 1b), recognized in South Kivu that causes a much more severe medical distress than the earlier strains. Following the July 2022 declaration, WHO deemed the situation a public health emergency of worldwide concern on August 14, 2024^5^. However, the DRC is not the only country affected by the outbreak; it has also spread to Burundi, Rwanda, and Uganda. The concerns regarding the potential for regional epidemics spreading further are being raised by the growing number of infections in these places^6^.

In addition to this, DRC is simultaneously also facing a major shortage of vaccination. While approximately 200,000 doses of the vaccine are available, the continent however requires over 10 million doses to address and control the mass expansion of the disease. Treatment options are extremely limited, such as the use of Tecovirimat, although researchers are still trying to determine its benefits^7^. Currently, most patients are treated with supportive care and pain management. One potential method to secure better resources for vaccines and treatments is to declare mpox as a public health emergency on a global scale, as was done by WHO on August 14, 2024, as multiple countries, including Pakistan, had reported cases of pox. Therefore, expanding the availability of vaccines, advancing research, and providing supporting care are essential components for a cumulative and an efficient response to the epidemic. This outbreak demands a coordinated and an immediate response for the effective management and suppression of the infectious disease outbreaks.

The CDC has assisted it in the effort to eliminate this disease in the last 15 years in DRC by supporting the provision of funds for the development of vaccines, establishing laboratory testing and training facilities while introducing training for the healthcare staff and assisting with diagnostics and sequencing. The government of the US has also taken measures by launching a response team to address the ongoing mpox outbreak in DRC with the provision of funds, healthcare workers and technical assistance. The testing strategy applied by the US healthcare system for diagnosis is the Non-variola Ortho-poxvirus (NVO) PCR test by the CDC. The strain is then analysed with the typical assays, specific to each clade^8^. There are numerous challenges that make it difficult to fight mpox, including a lack of treatment kits, testing facilities, and immunizations. However, the WHO and its partners are working to develop an effective immunization programme and establishing diagnostic tests and surveillance at DRC. Awareness programs for communicable diseases have also been initiated, including addressing the stigma related with mpox. They are also actively working with the DRC to strengthen their plans for vaccines and health security in the U.S.

Ultimately, due to the shortage of vaccines as we have mentioned before, there is tremendous pressure on the healthcare system, pertaining to the outbreak. Also, the new emerging class IIb, calls for the need to not only address but also scale up the production of vaccines and ensure that adequate supporting care is available.^9^ Moreover, if further therapies and medical attention is required in addition to the current treatments, research should be conducted expeditiously to add to the deficiency of the advised ministrations. Hence, to strengthen the response comprehensively, emphasis on encouraging public health workers to research on new antivirals and further treatment should be undertaken. Additionally, airport tests and increased wastewater monitoring, should be implemented. The testing rate should also be increased by establishing regional testing centres along with making mpox screening mandatory at airports (especially at international airports). As mentioned earlier, the availability of vaccine doses should be increased by collaborating with manufacturers in China, India and others to produce affordable vaccines, this will also ensure widespread administration of vaccines to impoverished families of DRC. Vaccination camps should be deployed in rural areas and necessary medicines and personal protective equipment (PPE) should be made available in outbreak zones, curtailing to the spread of the disease. Raising awareness via social media influencers, religious leaders and public health workers to launch awareness campaigns should also be put into action.

Furthermore, standardized reporting of mpox cases should be strictly maintained to monitor constant progress and make prompt strategical changes in targeting disease as needed. Incorporation of genetic sequencing in routine mpox surveillance can be implemented, this will help in rapidly identifying specific strain of mpox and track its prevalence. For strains which can spread from animal-to-human further investigations needs to take place to better understand its transmission and establish protocols to minimize its spread.

## Data Availability

Data and materials are not applicable
